# Enhancing health communication through virtual reality-based art therapy: an opinion

**DOI:** 10.3389/fpsyg.2024.1438172

**Published:** 2024-07-30

**Authors:** Zhongyu Shi, Yanyun Wang

**Affiliations:** Shanghai Film Academy, Shanghai University, Shanghai, China

**Keywords:** virtual reality, art therapy, health communication, media perception, health education

## 1 Introduction

Art practice promotes health, helps manage life problems, and prevents physical and mental health conditions (World Health Organization, 2019). Art therapy refers to therapist-led, art-based creative practices to improve client mental health (Malchiodi, 2011); it pertains to physical activity and positive mood, and facilitates healing and health education and communication. Artistic activities (e.g., dance) are considered therapy forms that emphasize experience participation, action-oriented technology, and creative expression. Virtual reality (VR) has improved convenience in artistic activities—reducing space, materials, and tools limitations; it promotes self-acceptance and mental health knowledge acquisition (Kaimal et al., 2020; Shamri Zeevi, 2021).

There are comprehensive reviews on art-based health communication and education research, showcasing that art helps spread understanding of health problems and the need for an art–public health link. One study reviewed art application in American community health communication projects, encompassing studies with different cultural groups and health-sensitive topics (e.g., depression; Sonke et al., 2021). It shows the usability of art to establish relationships with these groups, attention to health issues, and to form a health communication program. Art therapy also induces healthy emotional states during activities, difficult-to-achieve emotional states, and improves individuals' ability to conduct healthy self-reflection (van den Broek et al., 2011).

Health communication has changed significantly in the new media era (Riva et al., 2019); it utilizes communication evidence, strategy, theory, and creativity to advocate for behaviors and practices that enhance individual- and community-level health and wellbeing (Edgar and Volkman, 2012). Effective health publicity uses educational information with entertainment and mass media platforms. Health communication has been combined with art to provide accessible and operational health information (Robinson et al., 2014; Orji and Moffatt, 2018). Moreover, perception- and art-oriented health communication uses the unique feelings generated in aesthetic encounters for transformation (National Center for Health Statistics, 2020a,b; Sajnani et al., 2020). VR-based art therapy is an innovative method that provides healing environments for people with physical and mental health problems, complementing health communication activities.

Research on VR-based health communication is limited and has primarily focused on immersion and health information visualization functions. For example, visualizing food products' impact on the environment and health through VR supermarkets that display related information can help promote environmentally-friendly food choices (Smit et al., 2021). VR videos (e.g., hand-washing promotion videos) can help promote Korean students' willingness to engage in preventive health behaviors (Meijers et al., 2022). VR vaccine information can also increase vaccination intention and influenza transmission concerns (Lee et al., 2023). However, these studies targeted specific demographics, neglecting VR's diverse applications.

The progress and affordability of technology allowed VR application to art therapy. Numerous studies have used VR to examine behavior (Banakou and Slater, 2014), neural processing (Limanowski et al., 2017); treat phobia (Botella et al., 2017), post-traumatic stress disorder, anxiety (Goncalves et al., 2012), eating disorders (Clus et al., 2018); and offer pain management (Freeman et al., 2017). This article speculates that VR use in interventions effectively promotes the integration of mental health education and practical efforts, marking a shift of VR from its use in treatment to its use in communication and experience, making it a powerful driver of health–art integration into daily life. It comprehensively reviews studies on VR-based art therapy, classifies them, outlines the benefits of VR's artistic use for health, and evaluates VR-based art therapy's effectiveness for health communication purposes.

## 2 VR art therapy and its health communication potential

There are several categories of VR-based art therapy. Some provide participants with a diversified activity environment, like TiltBrush (i.e., allows drawing in a 3D environment). Using the acoustic response mode enables several people to simultaneously conduct painting activities in a shared VR environment, enhancing the collective perceived connection and affinity (Hacmun et al., 2021; Haeyen and Noorthoorn, 2021). VR can also simulate the traditional ceramic vase modeling, provide users with digital tools, and connect health education with traditional culture by providing authentic, culturally-meaningful experiences (Capece et al., 2023) VR can also help reach different disease groups and tackle the therapeutic needs of different age groups. Researchers have evaluated VR's effectiveness in neurological rehabilitation programs for children with attention-deficit/hyperactivity disorder, and improved the ecological effectiveness of treatment and children's participation by providing interactive experiences (Goharinejad et al., 2022; Zangiacomi et al., 2022). VR use as a screening and training tool for older adults' cognitive impairment helped screen for diseases and disseminate health information (Skurla et al., 2022). Individuals can use VR to participate in artistic creation activities at home. VR's artistic creation background and types draw attention to health and health awareness (Cavalcanti Barroso et al., 2022; Stevenson and Orr, 2013).

With this lack alongside mobile media's popularity, improvements in sensory participation during health treatment and education enhance the aesthetic experience of artistic activities (Sajnani et al., 2020) and improve health knowledge acceptance (Tao et al., 2021). Aesthetic experiences are “heightened, immersive, and particularly meaningful” and “important to us because they demonstrate the expressive power of life” (Parrish, 2007). Thus, VR provides aesthetic experiences and effectively realizes the integration of health education, artistic creation, and daily life. Public health professionals often mention the importance of art in cultivating a sense of meaning, and that meaningful experiences are more memorable and help people consider problems more deeply (Koch, 2017). The theoretical framework of experiential aesthetics includes artistic perception (impression side) and active artistic creation (expression side) (Koch, 2017). In VR's vivid aesthetic experiences, both artistic perception and creation are magnified by the immersive creative environment, realizing the concept of health knowledge through such experience. By integrating aesthetic experiences with health communication, VR can enhance the appeal, effectiveness, experience, and retention of health messages. This intersection of art, technology, and health communication opens innovative possibilities for health education and empowerment.

Health communication should be based on two-way information exchanges and use “common signs and behavior systems” (Malikhao, 2020), be approachable, and able to generate “feelings of mutual understanding and sympathy” between communication group members and audiences (Backer et al., 1992; Kreuter and Wray, 2003). Therefore, VR can be considered a practical alternative to traditional art therapy (Hacmun et al., 2021), and can be spread as a people-oriented care method. The creative connection afforded by VR basic creative art activities allows for the gradual elimination of communication barriers between health information and audiences (Haeyen et al., 2021; Gillibrand et al., 2023). VR may also be more attractive for some groups, such as people with mobility difficulties and youngsters with game addiction tendencies, expanding the range of audiences for health communication (Shamri Zeevi, 2021). Furthermore, health practitioners conducting artistic creation activities with audiences helps reduce the communicator–audience and doctor–patient power dynamics (Horghagen et al., 2007) producing positive psychological reactions on both sides. Using VR as an experiential tool in physical and mental healthcare activities stimulates interest and promotes communication and information dissemination.

## 3 Advantages and limitations

### 3.1 Advantages

Communication activities can be considered as aesthetic art. VR injects vitality into health communication by updating the experience of artistic healing and health activities and providing multiple simulation environments. By doing so, classic narratives can be effectively revitalized, transformed into dynamic narratives, and their rigidity can be challenged (Chinn and Kramer, 1991; Kaimal et al., 2020). The integration of physical interaction and VR-based artistic creation further improves (vs. traditional health communication) participation in artistic creation (Lohrius and Malchiodi, 2018), enabling involvement in health-related activities tailored to conditions, promoting health knowledge acquisition, and personalized narrative cultivation (Bale et al., 2023). Through its immersion and foresight, VR integrates entertainment and education into health communication.

VR technology has great appeal to youngsters, who are proficient in technology. They are often the promoters and audiences of VR-based health communication because of their openness to new technologies and comfortableness in using VR (Kouijzer et al., 2023). To maintain the relevance of health communication, VR contents are generally designed to be more enjoyable than traditional contents (Namkoong et al., 2023).

VR provides a safe experience for engagement in various artistic activities. People can experience the “sense of existence” of events through virtual interaction with objects (Rivera et al., 2015). While traditional official agency communication and one-way printed publicity only enable passive education, VR affords a sense of existence that can produce an embodied direct experience, enabling the application of own health understanding into artistic practice to obtain more intuitive and profound health knowledge. Some patients with mental illnesses, severe dementia, and risks of self-injury and aggression may consume non-edible objects and not be allowed to use tools to participate in manual creative activities (e.g., carpentry; Vaartio-Rajalin et al., 2021). VR may enable them to experience virtual creation while ensuring their safety (Kouijzer et al., 2023).

### 3.2 Limitations

VR-based art therapy use in health communication has several challenges. VR technology development has been uneven across fields, and yet VR-based art therapy mandates ongoing technical refinements to achieve universal applicability. Additional sensory inputs and actuators, like the amalgamation of olfaction and gustation (Spence et al., 2017), remain under development. Users often believe that art therapists are reluctant to embrace novel technologies due to either regional technological disparities or a lack of related proficiency (Haeyen, 2020). However, this perspective overlooks the potential advantages of VR-based art therapy.

## 4 Discussion

VR-based art therapy utilizes VR's sense of presence and immersion, has diversified practice forms, and promotes inclusiveness. Furthermore, while VR-based health communication provides passive educational experiences, simulation observation, and skill training, VR-based art therapy gives way for inspiration from artistic creation and self-expression. Since the promotion of health knowledge and emotional resonance sometimes overlap, health communication activities could use VR-based art therapy to customize the environment according to healthcare needs and encourage spontaneous expression. This personalized method may be more adaptable to participants' real-time reactions, making health communication more effective for each user. Thus, VR-based art therapy may be applicable to health communication.

This article presents the following opinions on VR-based art therapy application and research in health communication activities. First, empirical research should validate VR-based art therapy's effectiveness in health communication, specifically its effects on mental health, emotional release, and health behaviors. Research and feedback mechanisms should be established, and user behavioral and physiological reaction data related to health communication activities using VR-based art therapy should be collected for activity optimization. This will help us better understand the effect of different interventions and conduct the necessary adjustments. Notably, behavioral and physiological data can only be interpreted in light of patients' subjective assessment of therapy effectiveness and subsequent health-related behaviors.

Second, VR can be used to design personalized art experience programs (e.g., painting) to meet individual preferences. Various VR intervention types (e.g., VR experiences) should be distinguished to secure targeted treatment (Perez-Marcos, 2018).

Third, VR's ability to embody different perspectives should be leveraged to build empathy and self-awareness around health issues. VR-based artistic experiences tailored for health issues (e.g., mental health) should be designed, and VR's interactive features should be employed to stimulate emotional responses (e.g., experiences that show the treatment's emotional journey).

Fourth, VR can be used to create communal artistic endeavors that facilitate engagement, connections, and a platform for advocacy. Public health institutions should also incorporate VR-based reality art therapy equipment, as these can provide a platform for personal narrative exploration in health education. Allowing session-based artwork continuation could also deepen the engagement with health topics.

Fifth, interdisciplinary medical teams should cooperate with technical teams to design VR-based artistic health communication activities, ensuring that the activities meet professional and safety standards.

Finally, future research could quantify the benefits of VR-based art therapy in health communication and explore intervention optimization for different contexts. Studies on long-term outcomes and specific VR-based health communication intervention effectiveness in various demographic groups would provide deeper insights into VR's potential.

VR-based art therapy presents a promising frontier for enhancing health communication, as its immersive artistic environments support health activity simulation, aesthetic experiences, and participants' understanding—hence contributing to more effective health communication. As VR evolves, its art therapy application could become crucial in health communication for its immersion, convenience, and diversity.

## Author contributions

ZS: Conceptualization, Visualization, Writing – original draft. YW: Writing – review & editing.
